# The effect of ciprofloxacin on sperm DNA damage, fertility potential and early embryonic development in NMRI mice

**Published:** 2012

**Authors:** Fatemeh Zobeiri, Rajab-Ali Sadrkhanlou, Siamak Salami, Karim Mardani, Abbas Ahmadi

**Affiliations:** 1 *Department of Basic Sciences, Histology and Embryology Section, Faculty of Veterinary Medicine, Urmia University, Urmia, Iran;*; 2 *Department of Clinical Biochemistry and Nutrition, Faculty of Medicine, Urmia University of Medical Sciences, Urmia, Iran; *; 3 *Department of Food Hygiene and Quality Control, Faculty of Veterinary Medicine, Urmia University, Urmia, Iran.*

**Keywords:** Ciprofloxacin, Embryonic development, Fertilization, Mice

## Abstract

Side effects of ciprofloxacin (CPFX), a widely used broad spectrum antibiotic with fluoroquinolone core, have been reported in different organs. In the present study we sought to elucidate the impact of ciprofloxacin on sperm chromatin integrity and sperm DNA damage using Aniline Blue and Acridine Orange technique, respectively. The fertility potential in male mice was also evaluated. NMRI male mice of 8-week old were included in this study and they were randomly divided into three groups. The first group was received low dose (LD) of ciprofloxacin (206 mg kg^-1^, PO) and the second was treated with high dose (HD) of ciprofloxacin (412 mg kg^-1^, PO) for 45 consecutive days. The control mice were only treated with oral carboxymethyl cellulose for 45 consecutive days. Sperm cells were removed from cauda epididymis and analyzed for chromatin integrity and DNA damage. In addition, the rate of fertilization, two cell embryos, blastocysts, arrested embryos and their types was examined using zygotes cultured in human tubal fluid - bovine serum albumin (HTF-BSA) medium. Concomitant significant increase in DNA damage and protamine deficiency of the sperm cells in ciprofloxacin treated mice were observed (*P* < 0.05). In addition, the fertilization rate and embryonic development in treated mice were significantly lower than that of control mice, but the embryo arrest rate in treated mice was significantly higher than that of control group (*P* < 0.001). In conclusion CPFX was able to induce DNA damage and chromatin abnormalities of sperm cells which could be contributed in the observed low fertilization rate and retarded embryonic development.

## Introduction

Approximately 15-20% couples in the reproductive age are suffering from infertility in which male infertility is a contributory factor in half of all these couples.^1^^, ^^2^ Among the variety of causes, environmental factors such as chemical agents and drugs seem to be among the most important factors of infertility. Pharmacologically mediated male infertility would be associated with misleading drug administration, invalid dietary supplementation or recreational drug use. Many antibiotics have been reported to exert adverse effects on male fertility,^3^ however; there are few data on the majority of these medications. Fluoroquinolones (FQs) are a group of antibacterial agents that have been used routinely since 1980. The application of FQs is in treatment of urinary, skin, gastrointestinal, respiratory, bone and joint infections, as well as sexually transmitted diseases.^4^ Ciprofloxacin (CPFX) is a 4-fluoro-quinolone antibiotic commonly used in treatment of many microbial infections. The antibacterial mechanism of CPFX is based on the inhibition of the bacterial type II topoisomerase/DNA gyrase enzyme.^5^ The cross-reactivity of fluoroquinolones including CPFX with mammalian topoisomerase II has been reported previously.^6^^,^^7^ Studies related to the cytotoxic effects of CPFX revealed that this antibacterial agent inhibits the growth of various cultured mammalian cells.^8^^,^^9^ Cellular DNA damage and chondrotoxicity were reported to be induced by the CPFX.^10^^,^^11^ Impairment in testicular function and structure have been approved to be caused by CPFX .^12^^,^^13^ It was reported that the semen quality and embryonic development may be interrelated.^14^ Janny and Menezo have found that low cleavage rate and reduction in blastocyst percentage was correlated with sperm abnormality.^15^ Chromatin abnormalities and DNA damage can be considered as male subfertility indicator regardless of the routine indications of male infertility such as sperm concentration, motility and morphology.^16^

Given the paternal gametes affect embryonic development up to the blastocyst stage, any complement sperm parameter evaluation is of great importance.^17^ In the present study, DNA damage and chromatin integrity and their influences on infertility in an animal model have been investigated.

## Materials and Methods

**Animals and treatment groups. **8-week old male NMRI mice were used in this study. The mice were obtained from the animal resources center of the Faculty of Veterinary Medicine, Urmia University, Iran and kept under controlled environmental conditions (22 ± 2 °C, 30-60% relative humidity, 12/12h dark-light cycle). Following one week acclimatization, animals were randomly assigned to three groups each of four male mice as control-sham and test groups. All mice were fed *ad libitum* with a commercial mouse chow diet.

Ciprofloxacin (Fluka 17850, USA) was dissolved in 0.5 % carboxymethyl cellulose (CMC) and administered by gavage once daily for 45 consecutive days in doses of 206 mg kg^-1^ (low dose) and 412 mg kg^-1^ (high dose). The dose of 206 mg kg^-1^ for mouse is comparable to the human daily therapeutic dose, following correction for interspecies differences with a dose-scaling factor.^18^ CMC was given concurrently to control animals. At the end of the study period, the animals were euthanized by decapitation according to recommendation of the institutional ethical committee. Both epididymides (cauda and vas) of each mouse were transferred to a 60-mm Petri dish containing 1 mL of HTF-BSA (Sigma, USA) medium pre-warmed to 37 °C. The cauda was minced making 5-7 slashes with a 30-gauge needle of an insulin syringe. Then using forceps, the vas “walk down” to push out any remaining sperm. After 30 min incubation at 37 °C in an atmosphere of 5% CO_2_, the epididymal tissue was separated from the released spermatozoa.^19^ Then sperms were analyzed for chromatin integrity and DNA damage.

**Assessment of sperm single-stranded DNA. **Semen samples were washed three times in phosphate buffered saline (PBS). Thick smears of washed spermatozoa were prepared on pre-cleaned degreased slides and allowed to air-dry for 10 min. The smears were fixed for 1 h in ethanol-acetone (1:1) at 4 °C and allowed to dry for a few minutes before staining with Acridine Orange (AO) (0.19 mg mL^-1^) for 7 min at room temperature. After staining, the slides were gently rinsed in a stream of distilled water and air dried.^20^ This was followed by evaluation under fluorescence microscope (Model GS7, Nikon, Japan) with a 100 oil immersion objective. The percentage of spermatozoa stained with Acridine Orange was determined by counting 200 spermatozoa per slide. The monomeric AO, bound to normal double-stranded DNA, produces a green fluorescence, whereas the aggregated AO on single-stranded DNA yields a yellow to red fluorescence.

**Assessment of sperm chromatin integrity. **The Acidic Aniline Blue (AAB) stain specifically reacts with lysine residues in nuclear histones and reveals differences in the basic nuclear protein composition of the sperm. Histone-rich nuclei of immature sperms are rich in lysine and will consequently take up the blue stain. On the other hand, protamine rich nuclei of matured spermatozoa are rich in arginine and cysteine and contain relatively low levels of lysine, which means they will not be stained by AAB.^20^ The air-dried fixed smears were stained for 7 min with 0.5 % Aniline Blue in PBS buffer. The pH was adjusted to 3.5 using acetic acid. Slides were gently rinsed in distilled water and air dried.^20^

**Oocyte collection. **Female mice of 6-8 weeks old were superovulated with pregnant mare^’^s serum gonadotrophin (PMSG: Folligon, Holland) (7.5 IU, IP) and human chorionic gonadotrophin (hCG: Folligon, Holland) (7.5 IU, IP) 48 h apart. At 13 hours post-hCG administration, female mice were sacrificed by cervical dislocation. Both oviducts of each female were transferred to a Petri dish containing 2.0 mL, HTF-BSA medium. Using a stereomicroscope, the swollen ampulla was found and oocytes dissected out for in vitro fertilization (IVF).^19^

**Sperm preparation and IVF. **Approximately 12-13 h after hCG injection of the female mice, the male mice were euthanized. Samples of each group were prepared from the sperm suspension as described in animal and treatment groups section. Spermatozoa were obtained by swim-up and capacitated by incubating at 37 °C under 5% CO_2_ for at least 1 h. Then sperm with the concentration of 1×10^6 ^total sperm per mL was added to 500 µL fertilization drop of HTF-BSA medium containing oocytes from three females. After four to six hours of incubation at 37 °C under 5% CO_2_, the cumulus cell free fertilized oocytes were transferred to fresh drops of HTF-BSA medium for culture of embryos. All of the medium droplets were covered with mineral oil.^21^

**Assessment of fertilization. **Fertilized oocytes were evaluated by appearance of the pronuclei and polar bodies under the inverted microscope with magnification of 200×.

**Assessment of embryonic development. **About 24 h after the zygotes culture, the two cell embryos rate was assessed and in vitro embryonic development was evaluated at 120 h under phase-contrast microscopy. The intact, fragmented and/or lysed embryos which did not develop were recorded as “arrested embryos”.

In this experiment the rate of cell lyses was divided into three following categories:

Type I: fully lysed, necrotic and/or fragmented embryos. Type II: embryos with partially lysed/ fragmented blastomeres. Type III: embryos with some lysed/fragmented blastomeres and/or cytoplasmic vesicles.^22^

**Statistical Analysis. **Statistical analyses of sperm parameters were carried out using one-way ANOVA followed by Tukey test using SPSS software Ver. 16 (SPSS, Inc., IL, USA) and assessment of the results of IVF was analyzed using two proportion comparison in Minitab software version 15.1 (Minitab Inc., PA, USA).

## Results

**DNA damage and chromatin integrity of sperms. **The level of abnormal single-stranded sperm DNA in CPFX-treated mice was significantly higher than that of control group (*P* < 0.05). There was no significant difference between LD and HD treated-mice (*P* > 0.05). The percentage of sperms with protamine deficiency in treated groups (LD and HD) was higher than those of control group (*P* < 0.05). Also, there was a significant difference between LD and HD (*P* < 0.05) (Table1).

**Table 1:** Effects of CPFX on DNA integrity and chromatin quality. Data are presented as mean ± SEM.

**Groups**	**Positive Acridine Orange staining (%)**	**Positive Aniline Blue staining (%)**
**Control**	8.75 ± 2.01^a^	9.37± 0.31^a^
**Low dose**	29.25 ± 1.37^b^	19.50 ± 0.28^b^
**High dose**	31.50 ± 2.62^b^	25.75 ± 0.85^c^

abc Different letters in each column indicate significant differences (*P* < 0.05).

**Fig. 1:**
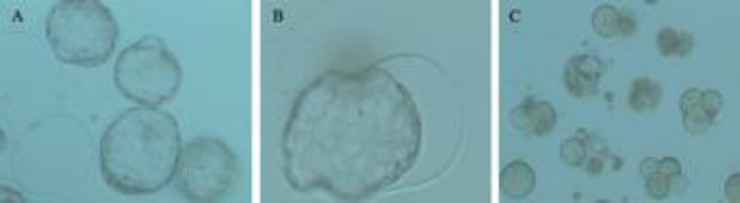
*In vitro* development of embryos at 120 h of culture. A. Control group. Differentiation to expanded and hatched blastocysts B. Control group, blastocyst in hatching stage. C. Treatment groups. Embryos arrested and type I, II and III of embryo quality

**Fertilization rate and embryonic development. **The results of fertilization rate and embryonic development in different groups are summarized in Table 2. The data show that fertilization rate and two cell embryos rate in the groups of mice treated with CPFX were significantly lower than of the control group (*P* < 0.001) but the rate of two cell embryos in low dose treated-mice was not significantly lower than that of the controls. Percentage of embryos in blastocyst stage after culturing for 120 h in the control group was significantly higher than that of CPFX treated groups (*P* < 0.001) (Table 2, Fig. 1 A and B).

There was a marked increase in percentage of arrested embryos type I, II and III in CPFX treated mice in comparison with the control group (*P* < 0.001) (Fig. 1C). Furthermore, embryonic arrest type I was more frequent in treated groups than in control (*P* < 0.001).

**Table 2:** The number and (percentage) of oocytes, fertilized oocytes, embryos (two cells and blastocysts) in groups.

**Groups**	**Oocytes**	**Fertilized oocytes**	**Two cells**	**Blastocysts**	**Arrest**	**Arrest type I**	**Arrest type II**	**Arrest type III**
**Control**	123	101 (82.11)^a^	100 (99.00)^a^	82 (81.18)^a^	19 (18.81)^a^	2 (1.98)^a^	7 (6.93)^a^	10 (9.90)^a^
**Low dose**	229	144 (62.88)^b^	140 (97.22)^a^	31 (21.52)^b^	113 (78.47)^b^	46 (31.94)^b^	31 (21.52)^b^	36 (25.00)^b^
**High dose**	345	211 (61.15)^b^	148 (70.14)^b^	43 (20.37)^b^	168 (79.62)^b^	75 (35.54)^b^	47 (22.27)^b^	46 (21.80)^b^

abc Different letters in each column indicate significant differences (*P* < 0.001).

## Discussion

Our findings showed that after CPFX administration, the percentage of immature sperms (sperms with protamine impairment) and sperms with single stranded DNA (DNA damaged sperms) were increased significantly. According to previous reports, sperm DNA damage and nuclear chromatin abnormalities occur during impaired spermiogenesis which in turn results into remarkable abnormality in DNA packing.^23^ Consequently, free radicals especially reactive oxygen species (ROS) induce severe DNA damges^24^ and/or lead to cellular apoptosis.^25 ^Our light microscopic analyses showed that the percentage of sperms with nuclear immaturity increased in CPFX-treated groups. Therefore, we came close to this fact that CPFX induced detrimental effect on sperm chromatin packing and consequently elevated DNA damage. 

Abnormal endogenous nicks in DNA, malfunction, and/ or any damages of nucleases are two different theories for abnormal chromatin packing.^26^^-^^28^ As CPFX is known as topoisomerase inhibitor, thus we can conclude that CPFX impairs creating and ligating nicks in DNA which in turn blocks protamination and consequently induces internal DNA damage by preventing its repair and increasing its susceptibility to damage.^29^ This theory has been proofed with elevated DNA damage in sperms after CPFX administration. Accordingly, higher percentage of the sperms underwent DNA damage in low and high dose CPFX-treated animals in comparison with sperms from control group (Table 1). The notable point was that, the DNA damage was not differed significantly in low and high dose CPFX-treated animals. Thus, it comes close that CPFX is able to induce its pathological effect in low dose.

In addition to DNA damage resulted from impaired protamination, Herold *et al.* and Jun *et al*. showed that CPFX is able to increase caspase 8 and 9 synthesis in order to start apoptosis process.^9^^,^^30^ It was also showed that mitochondrial dysfunction severely elevates nuclear DNA fragmentation in sperms.^31^ Considering the findings of the present study (increased DNA damage), it can be concluded that CPFX not only elevated DNA damage by abnormal protamination but also it may begin apoptosis by increasing caspase 8 and 9 synthesis and/or inducing pathological impact on mitochondrial function.^32^ Further analyses are needed to clarify the exact mechanism of DNA damage in CPFX treated mice.

We also found that the fertilization rate and embryonic development in the CPFX-treated mice were lower than those of the control group. It is well known that, there is a positive correlation between the quality of the sperms with embryonic development in both *in vivo* and *in vitro*.^17^^,^^33^ The ability of the embryo to survive appears to be negatively correlated with the level of DNA fragmentation in the germ line.^34^ Identified evidences of the present study suggested that disturbances in the organization of the CPFX-induced sperms genome, pathologically affected their fertilizing potential. Accordingly the percentage of arrested embryos was elevated significantly (*P *< 0.001) after CPFX administration (Table 2). Similar to sperm DNA damage the results for IVF confirmed that the CPFX exerted its adverse effect in low dose. The IVF outcomes for both low and high dose treated animals were not statistically significant. 

It has been shown that protamine deficiency and increased histone remnants in sperms as a result of any exposure with exogenous pathogens such as topoisomerase inhibitors have resulted in lower fertilizing ability and embryonic development.^35^ In the case of such impairment, the replacement of histone proteins by protamines will be affected severely. Therefore, this impaired replacement will disable DNA to properly decondense after entering into oocyte (which is very essential in order to perform fertilization process).^36^ As CPFX is known as topo-isomerase inhibitor, thus, we can conclude that protamine deficiency in CPFX-exposed group not only reduced IVF outcome by increasing DNA damage but also diminished DNA decondensing after entering into oocyte. Ultimately the percentage of blastocysts was decreased in both low and high dose CPFX-administrated animals (Fig. 1C). 

In conclusion, current findings illustrated that CPFX induced its detrimental effects by protamine impairment which was able to increase DNA susceptibly for damages and disable DNA decondensing following fertilizing the normal oocytes. Therefore, the sperms in low and high dose CPFX-administrated animals showed significantly lower IVF outcome in comparison with those of controls.
